# Impact of Dentine Pretreatment with Matrix Metalloproteinase Inhibitors on Bond Strength of Coronal Composite Restorations: A Systematic Review and Meta-analysis of *In Vitro* Studies

**DOI:** 10.1055/s-0042-1757582

**Published:** 2022-11-18

**Authors:** Hasan Jamal, Rayan Yaghmoor, Hassan Abed, Anne Young, Paul Ashley

**Affiliations:** 1 Paediatric Dentistry Department, Eastman Dental Institute, University College London, London, United Kingdom; 2 Department of Biomaterials and Tissue Engineering, Royal Free Hospital, UCL Eastman Dental Institute, London, United Kingdom; 3 Department of Microbial Diseases, UCL Eastman Dental Institute, Royal Free Hospital, London, United Kingdom; 4 Department of Restorative Dentistry, Faculty of Dentistry, Umm Al-Qura University, Makkah, Saudi Arabia; 5 Department of Basic and Clinical Oral Sciences, Faculty of Dentistry, Umm Al-Qura University, Makkah, Saudi Arabia

**Keywords:** restorations, bond strength, matrix metalloproteinase inhibitors, enzymes, caries, dentine.

## Abstract

Matrix metalloproteinase (MMP) enzymes participate in collagen matrix degradation, including in dentine, potentially compromising bond strength. Therefore, MMP inhibitors have been hypothesized to improve restoration bond strength and stability. This systematic review aimed to evaluate the influence of different MMP inhibitors applied as dentine surface pretreatments on the immediate (24 hours) and longer term (months) bond strength of direct coronal composite restorations. This systematic literature review followed the Preferred Reporting Items for Systematic Review and Meta-analyses (PRISMA) statement. A systematic literature search of three databases (Ovid MEDLINE, Ovid Embase, and Google Scholar) was conducted independently by two reviewers from inception to April 2022. An adapted quality assessment tool was independently applied by two reviewers for risk of bias assessment. RevMan v5.4 software was used for meta-analyses. A randomeffectsmodel was used to generatemean differences with 95% confidence intervals for treatment and control comparisons. The Q-test and I2-test were used to test for heterogeneity. The proportion of total variance across studies attributable to heterogeneity rather than chance was calculated. Overall effects were tested using the Z-test, while subgroup differences were tested using Chi-squared tests. Of 934 studies, 64 studies were included in the systematic review and 42 in the meta-analysis. Thirty-one MMP inhibitors were reported, three of which were included in the meta-analysis: 2% chlorhexidine (CHX), 0.3M carbodiimide (EDC), and 0.1% riboflavin (RIBO). Pretreatment with 2% CHX for 30 and 60 seconds did not significantly improve bond strength compared with controls either immediately or after long-termageing. However, pretreatment with 0.3MEDC and 0.1% RIBO (but not CHX) significantly improved bond strength compared with control groups both immediately and over time. Most studies showed a medium risk of bias. These in vitro findings pave the way for rationale clinical trialing of dentine surface pretreatment with MMP inhibitors to improve clinical outcomes.

## Introduction

Since their introduction around six decades ago, restorative adhesives have undergone numerous improvements. 1 2 Despite these advances, adhesive restorations often lose their bond strength over time, leading to their failure. 3 4 Adhesive restorations critically rely on their bond with the tooth structures for strength, with the interface—the hybrid layer—crucial in determining the bond's longevity and stability. 5 6 The collagen fibrils in dentine (mainly type 1 collagen) are key to establishing a strong bond, and their deterioration is thought to be the main reason underlying bond failure to dentine. 7

Recent studies have examined the role of endogenous enzymes present within the dentine extracellular matrix (ECM) and their effect on bond stability. Among these enzymes, matrix metalloproteinases (MMPs) represent a group of calcium- and zinc-dependent host-derived enzymes. 8 MMPs are divided into six subgroups: collagenases (MMP-1 and MMP-8), stromelysins (MMP-3, MMP-10, MMP-11, and MMP-20), gelatinases or type-IV collagenases (MMP-2 and MMP-9), matrilysin (MMP-7), metalloelastase (MMP-12), and membrane-type metalloproteinases (MMP-14, MMP-15, MMP-16, and MMP-17). 9 Of these, four MMPs have been identified within the dentine extracellular matrix: MMPs-2, -8, -9, and -20, with MMP-2 and -9 as the most abundant. 10 11 These enzymes are secreted by odontoblasts during odontogenesis and remain silenced and inactive within the dentine ECM. 12 However, these MMPs are activated either by biological acids produced by cariogenic bacteria 13 or acids introduced during acid etching. 14 15 When activated, they start to degrade the exposed collagen fibrils within the dentine. 16 Therefore, inhibiting MMPs could help to preserve the hybrid layer and, therefore, bond stability.

Several types of MMP inhibitor (synthetic and natural) have been described including benzalkonium chloride, 17 18 chlorhexidine, 18 19 20 21 galardin, 22 green tea extract, 23 24 and zinc. 25 MMP inhibitors can be administered either as dentine surface pretreatments or those incorporated into the adhesive. The current systematic review and meta-analysis aimed to collect and analyze the available *in vitro* evidence on the influence of different MMP inhibitors applied as dentine surface pretreatments on the immediate and long-term bonding strength of coronal composite restorations. The null hypothesis was that there would be no difference in bond strength after MMP inhibitor use compared with controls.

## Methods

### Eligibility Criteria

The systematic review was developed according to the PICO scheme ( Table 1 ) 26 and was conducted according to the Preferred Reporting Items for Systematic Review and Meta-analyses (PRISMA) guidelines 27 :

**Table 1:** Keywords and the strategy used in MEDLINE and Embase

	Medline (Ovid)	Embase
P	1. Extracted human teeth.mp./OR Human teeth.mp. 2. Sound dentine.mp./OR healthy dentine.mp. 3. Carious affected dentine.mp./OR Caries affected dentine.mp./OR affected dentine.mp. 4. Dentine$.mp. 5. 1 OR 2 OR 3 OR 4	1. Extracted human teeth.mp./OR Human teeth.mp. 2. Sound dentine.mp./OR healthy dentine.mp. 3. Carious affected dentine.mp./OR Caries affected dentine.mp./OR affected dentine.mp. 4. Dentine$.mp. 5. 1 OR 2 OR 3 OR 4
I	6. Matrix metalloproteinase inhibitors/OR MMP inhibitors.mp.	6. Matrix metalloproteinase inhibitors/OR MMP inhibitors.mp.
C	7. No matrix metalloproteinase inhibitors/OR No MMP inhibitors	7. No matrix metalloproteinase inhibitors/OR No MMP inhibitors
O	8. Bond strength/OR Bond stability	8. Bond strength/OR Bond stability
Combined	1 OR 2 OR 3 OR 4 AND 6 AND 7 AND 8)	1 OR 2 OR 3 OR 4 AND 6 AND 7 AND 8)

Abbreviation: MMP, matrix metalloproteinase.

Population: all studies examining extracted human teeth, caries-free dentine, healthy dentine, sound dentine, carious-affected dentine, or affected dentin.

Interventions: all studies examining MMP inhibitors as dentine surface pretreatments prior to direct coronal composite restoration placement. Therefore, studies that used luting cements and glass ionomer cements were excluded.

Comparator(s)/control(s): teeth without intervention (i.e., without the addition of MMP inhibitor). Studies that included no comparator were excluded.

Outcome: the main outcome was bond strength or bond stability at the microscale (by microtensile and microshear testing). Studies that tested bond strength at the macroscale were excluded. Included studies needed to have aged the samples for at least 24 hours in water or artificial saliva. Thus, studies with ageing up to 24 hours only and/or studies that used ageing solutions other than water or artificial saliva were excluded.

### Search Strategy

#### Types of Searched Studies

The search included published, peer-reviewed *in vitro* studies presenting the results (means and standard deviations [SDs]) quantitively and numerically in the English language. Thus, studies that reported the results in graphs or figures only were excluded. Non–peer reviewed studies, conference posters, letters, theses, reviews, and editorials were excluded.

#### Period of Reviews (Timing) and Databases

A systematic literature search was conducted in three databases: Ovid MEDLINE (1946–April 2022), Embase (1974–April 2022; see Table 1 ), and Google Scholar (up to April 2022).

With respect to the search strategy for Google Scholar, the following terms were used: “Extracted human teeth” OR “human teeth” OR “Sound dentine” OR “healthy dentine” OR “affected dentine” OR “Carious affected dentine” OR “Caries affected dentine” OR “Dentine” AND “Matrix metalloproteinase inhibitors” OR “MMP inhibitors” AND “Bond strength” OR “Bond stability.”

### Data Selection and Collection Processes

Full texts of all eligible studies were uploaded to reference management software (EndNote X9.3.1) and duplicate publications were removed automatically. Two authors (H.J. and R.Y.) screened the titles and abstracts, and the full text of studies meeting the inclusion criteria was read. Two evaluators (H.J. and R.Y.) independently screened each full-text paper based on the eligibility criteria. In case of discrepancies about study eligibility between the two reviewers, a further evaluator was involved (H.A. or P.A.). A data extraction form included the following: authors' names, year of publication, type of MMP inhibitor used, duration of MMP inhibitor used as dentine pretreatment, substrate condition, type of bonding agent, type of ageing solution, period of ageing, type of bond strength test, and bond strength means. Two reviewers (H.J. and R.Y.) were independently involved in data collection. An experienced third reviewer (P.A.) independently extracted data from 10% of studies to check process consistency. Conflicts of opinion were resolved through consensus by consulting a further reviewer (H.A. or A.Y.).

### Risks of Bias and Quality Assessment

A quality assessment tool adapted from a previous study 28 was independently used by two reviewers (H.J. and R.Y.). The tool evaluated bias in terms of sample randomization, substrate condition, duration of dentine pretreatment, the use of materials according to the manufacturer's instructions, storage medium, interface surface area, restorative and bond tests performed by a single operator, sample size calculation (power analysis), and blinding of the operator during bond strength testing. Minor modifications were added to the risk of bias evaluation tool, which are “dentine pretreatment duration” and “storage medium”. For each component of the tool, the letter “Y (yes)” was assigned if the author reported the item and “N (no)” if it was not reported. The grading judgement of “low,” “medium,” or “high” for the study was based on the total number of “Ys” as follows: one to five (high), six, or seven (medium), and eight or nine (low).

### Data Synthesis

Findings were summarized narratively using text and tables. For example, findings were summarized according to type of MMP inhibitor used, duration of dentine pretreatment, substrate condition (caries-free or caries-affected), type/mode of bonding agent, type of ageing solution, period of ageing, type of bond strength test, and mean bond strength.

### Meta-analysis

Review Manager (RevMan) version 5.4 software from the Cochrane Collaboration was used for meta-analyses using the following information: the average difference in outcome measures between the intervention and control groups, the number of teeth in each treatment group, and the standard deviations. These data were categorized into three time periods: 24 hours, 6 months, and 12 months, where applicable, and further divided into the type of MMP inhibitor, the adhesive application method used (self-etching or etch and rinse), and the pretreatment duration. Only MMP inhibitors applied for 30 and 60 seconds were included as they contained enough data for the meta-analysis.

The mean differences (MDs) and their 95% confidence intervals (CIs) were calculated. Findings from all comparisons were generally pooled according to the three time periods (24 hours, 6 months, and 12 months). After establishing the pooled MDs according to time, additional pooling was carried out depending on the various parameters indicated. A positive MD supports the experimental group, whereas a negative MD favors the control group. A random-effects model was used to generate MDs with 95% CIs for treatment and control comparisons.

The *Q* -test and *I ^2^* -test were used to test for heterogeneity. The *I ^2^* statistics was interpreted according to the Cochrane guidelines, with 0 to 29% as being low, 30 to 50% as moderate, and 50 to 90% as considerable heterogeneity. 29 The proportion of total variance across studies attributable to heterogeneity rather than chance was calculated. Finally, the overall effects were tested using the *Z* -test, while subgroup differences were tested using Chi-squared tests.

The following analyses were carried out:

1. 2% chlorhexidine (CHX) versus control at baseline (24 hours).
2. 2% CHX versus control at 6 months.
3. 2% CHX versus control at 12 months.
4. 0.3 M 1-Ethyl-3-(3-dimethyl aminopropyl) carbodiimide (EDC) versus control at baseline.
5. 0.3 M EDC versus control at 12 months.
6. 0.1% riboflavin (RIBO) versus control at baseline.
7. 0.1% RIBO versus control at 6 months.
8. 2% CHX versus control at baseline (according to pretreatment duration of 30 seconds).
9. 2% CHX versus control at 6 months (according to pretreatment duration of 30 seconds).
10. 2% CHX versus control at baseline (according to pretreatment duration of 60 seconds).
11. 2% CHX versus control at 6 months (according to pretreatment duration of 60 seconds).

## Results

### Study Selection

**Fig. 1:**
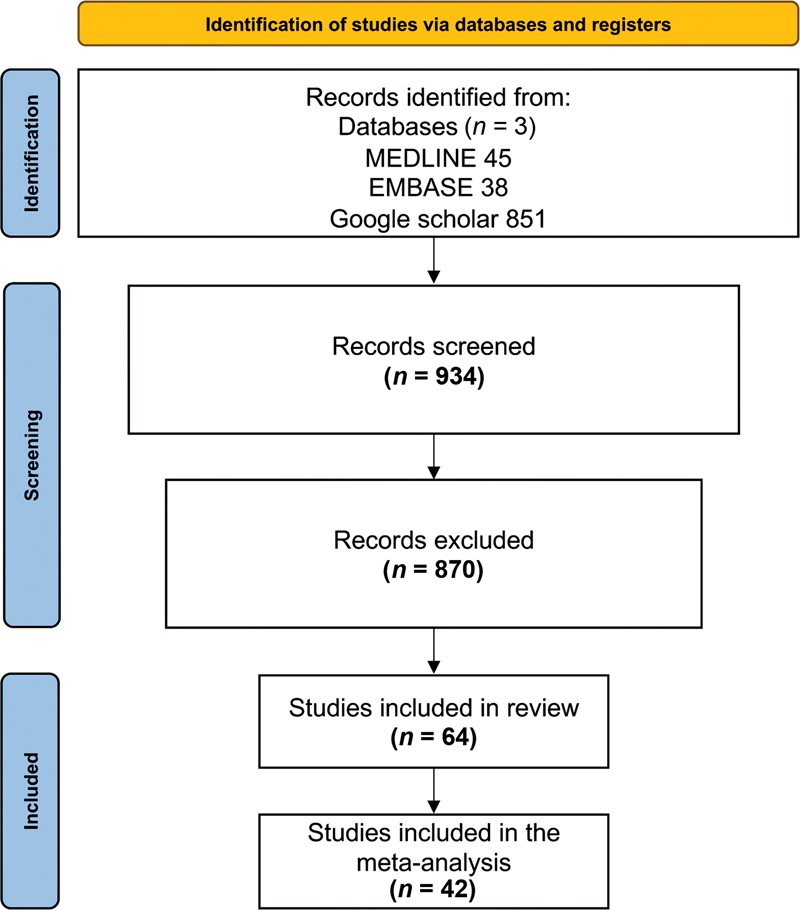
PRISMA 2020 flowchart diagram of study selection. 27 PRISMA, Preferred Reporting Items for Systematic Review and Meta-analyses.

A flowchart summarizing the selection process according to the PRISMA statement is shown in Fig. 1 . 27 During the initial search, 934 potentially eligible studies were retrieved. After removal of duplicates, 763 studies remained of which 193 remained after reviewing the titles and 163 after reviewing the abstracts. Following reading the full texts, 64 studies were included in the study and 42 were included in the meta-analysis.

### Study Characteristics

The data obtained from the included publications are listed in Table 2 . The 64 included *in vitro* studies were published between 2009 and 2022.

**Table 2:** Characteristics of the included studies

Study/year	MMP inhibitor type	Pretreatment duration (s)	Substrate condition	Adhesive system (mode of application)	Ageing solution	Period of ageing	Groups	Bond strength means (SD)
Baena et al/2020 30	CS	60	Caries-free	Optibond FL (Kerr; etch-and-rinse; OFL) Scotchbond Universal (3M; self-etch; SBU)	Artificial saliva	24 hours	CS 0.1% + OFL Control (OFL) CS 0.1% + SBU Control (SBU)	38.0 (7.7) 41.3 (14.5) 28.1 (14.3) 25.0 (16.5)
						10,000 thermocycles	CS 0.1% + OFL Control (OFL) CS 0.1% + SBU Control (SBU)	29.2 (14.1) 32.2 (12.9) 33.1 (17.0) 30.4 (11.8)
Balloni et al/2017 31	CHX	60	Caries-free	Clearfil SE bond (self-etch)	Water	24 hours	CHX 2% Control	19.24 (11.89) 12.67 (7.43)
						6 months	CHX 2% Control	11.97 (9.95) 10.22 (5.00)
Bravo et al/2017 32	CHX	20	Caries-free	Adper Scotchbond 1XT (etch-and-rinse; ASB) Adper prompt (self-etch; AP) Single Bond Universal (self-etch; SBU)	Water	3 days	CHX 2% + ASB Control (ASB) CHX 2% + AP Control (AP) CHX 2% + SBU Control (SBU)	26.28 (9.29) 28.56 (5.83) 24.21 (7.52) 20.14 (4.87) 28.43 (9.78) 29.24 (7.90)
						3 months	CHX 2% + ASB Control (ASB) CHX 2% + AP Control (AP) CHX 2% + SBU Control (SBU)	32.26 (10.33) 19.82 (7.65) 28.51 (13.18) 20.86 (6.13) 44.11 (12.09) 23.54 (12.09)
						6 months	CHX 2% + ASB Control (ASB) CHX 2% + AP Control (AP) CHX 2% + (SBU) Control (SBU)	31.73 (5.18) 23.39 (5.69) 27.37 (4.40) 20.51 (5.66) 36.88 (6.65) 23.62 (7.07)
de Faria Teixeira et al/2015 33	CHX	60	Not mentioned	Clearfil SE bond (self-etch)	Water	24 hours	CHX 2% Control	28.0 (8.4) 24.2 (7.2)
						6 months	CHX 2% Control	33.4 (9.3) 21.8 (7.3)
Comba et al/2020 34	DCC	60	Caries-free	Scotch bond universal (SBU) (self-etch) and etch and rinse	Artificial saliva	24 hours	0.5M DCC SBU (ER) Control SBU (ER) 0.5M DCC SBU (SE) Control SBU (SE)	46.0 (5.3) 37.1 (12.5) 39.4 (11.1) 26.3 (11.4)
						12 months	0.5M DCC SBU (ER) Control SBU (ER) 0.5M DCC SBU (SE) Control SBU (SE)	33.5 (13.9) 31.0 (11.0) 35.3 (13.9) 13.4 (9.1)
Czech et al/2019 24	CHX EGCG	60	Caries-affected	Adper Single Bond 2 (etch and rinse)	Water	24 hours	EGCG 200 μg/mL CHX 2% Control	24.08 (7.20) 14.64 (7.74) 23.43 (7.73)
						6 months	EGCG 200 μg/mL CHX 2% Control	18.67 (8.51) 11.20 (4.79) 16.28 (9.58)
						12 months	EGCG 200 μg/mL CHX 2% Control	16.77 (5.50) 10.17 (3.02) 14.91 (6.92)
Dávila-Sánchez et al/2020 35	QUE HES RUT NAR PAC	60	Caries-affected	Scotchbond Universal (3M; etch and rinse)	Water	24 hours	HES 6.5% PAC 6.5% QUE 6.5% NAR 6.5% RUT 6.5% Control	18.41 (5.30) 20.66 (3.92) 24.58 (4.90) 24.64 (3.70) 26.00 (5.51) 14.42 (4.43)
						25,000 thermocycles	HES 6.5% PAC 6.5% QUE 6.5% NAR 6.5% RUT 6.5% Control	15.73 (6.07) 17.20 (2.72) 12.02 (5.21) 22.12 (2.92) 21.08 (4.75) 9.43 (4.29)
Costa et al/2019 36	CHX EGCG	60	Eroded (ERO) and non-eroded (non-ERO)	Clearfil SE bond (self-etch)	Water	24 hours	CHX 2% (non-ERO) CHX 2% (ERO) EGCG 0.1% (non-ERO) EGCG 0.1% (ERO) Control (non-ERO) Control (ERO)	40.87 (10.23) 49.30 (9.42) 53.67 (6.10) 61.61 (3.17) 52.44 (8.47) 59.25 (5.91)
						6 months	CHX 2% (non-ERO) CHX 2% (ERO) EGCG 0.1% (non-ERO) EGCG 0.1% (ERO) Control (non-ERO) Control (ERO)	32.77 (10.67) 36.91 (9.88) 50.02 (3.42) 44.63 (13.26) 47.64 (11.67) 45.16 (11.87)
El Baz and Aboulenien/2018 37	EGCG	60	Caries-free	Primer and Bond one (Dentsply; etch and rinse)	Water	24 hours	EGCG 0.1% Control	18.8 (0.2) 15.4 (0.7)
						6 months	EGCG 0.1% Control	17.6 (0.3) 12.2 (0.9)
						5,000 thermocycles	EGCG 0.1% Control	22.1 (0.7) 8.8 (0.8)
Fang et al/2017 38	MAP GM6001	60	Caries-free	Gluma Comfort Bond (etch and rinse)	Water	24 hours	MAP 1 mg/mL GM6001 10μM Control	19.31 (4.48) 18.86 (4.2) 19.25 (4.21)
						2,500 thermocycles	MAP 1 mg/mL GM6001 10μM Control	12.22 (4.49) 10.87 (4.27) 6.08 (3.12)
Fernandes et al/2021 39	CHX EGCG	60	Caries-free	Clearfil SE Bond Primer (self-etch)	Artificial saliva	24 hours	CHX 2% EGCG 0.01% Control	44.16 (6.81) 42.76 (7.36) 40.65 (6.51)
						12 months	CHX 2% EGCG 0.01% Control	33.58 (10.49) 34.91 (7.84) 33.85 (9.27)
Fialho et al/2019 40	CHX EGCG	60	Caries-affected	Adper Single Bond 2 (3M; etch and rinse)	Water	24 hours	EGCG 0.2% EGCG 2% EGCG 0.5% CHX 2% Control	32.65 (9.97) 29.16 (11.52) 28.57 (6.30) 33.33 (11.26) 35.81 (8.25)
						12 months	EGCG 0.2% EGCG 2% EGCG 0.5% CHX 2% Control	22.75 (9.38) 17.15 (10.61) 23.65 (7.19) 19.98 (7.01) 26.17 (12.28)
Gerhardt et al/2016 41	CHX EGCG GT	60	Caries-free	Clearfil SE Bond (self-etch)	Water	24 hours	CHX 2% EGCG 2% GT 2% Control	13.31 (3.36) 6.93 (3.43) 10.60 (4.69) 8.64 (5.52)
						6 months	CHX 2% EGCG 2% GT 2% Control	11.09 (4.98) 15.96 (5.32) 17.82 (12.20) 16.69 (7.20)
Campos et al/2019 42	CHX	Not mentioned	Caries-affected	Clearfil SE Bond (self-etch)	Water	24 hours	CHX 2% Control	19.84 (8.11) 24.89 (9.44)
						12 months	CHX 2% Control	17.59 (8.85) 28.30 (11.54)
Giacomini et al/2020 43	CHX	30	Caries-free	Adper Single Bond 2 (etch and rinse) Adper Single Bond Universal (etch and rinse) Adper Single Bond Universal self-etch (self-etch)	Artificial saliva	24 hours	CHX 2% (ASB) Control CHX 2% (ASU-ER) Control CHX 2% (ASU-SE) Control	28.41 (7.64) 33.35 (9.01) 33.66 (7.79) 31.62 (8.20) 37.47 (10.68) 45.62 (12.39)
						6 months	CHX 2% (ASB) Control CHX 2% (ASU-ER) Control CHX 2% (ASU-SE) Control	31.55 (6.15) 32.59 (9.44) 33.79 (6.24) 32.05 (7.04) 34.25 (11.21) 40.15 (14.77)
Grandizoli and Pinheiro/2018 44	CHX	60	Caries-affected	Clearfil SE bond (self-etch)	Water	24 hours	CHX 2% Control	21.7 (16.3) 19.3 (11.9)
						6 months	CHX 2% Control	1.9 (1.8) 2.5 (1.2)
Karrabi and Danesh Kazemi/2016 45	CHX	120	Caries-free	Adper Single Bond (etch and rinse)	Artificial saliva	6 months	CHX 2% Control	52.67 (6.86) 28.84 (6.23)
Kasraei et al/2017 46	RIBO	120 Light activation	Caries-free	Adper Single Bond (etch and rinse)	Water	5,000 thermocycles	RIBO 0.1% Control	12.79 (3.64) 12.64 (2.35)
Lenzi et al/2014 47	CHX	60	Caries-free and caries-affected	Adper Single Bond (etch and rinse)	Water	24 hours	CHX 2% Control CHX 2% (CA) Control (CA)	32.8 (3.8) 30.7 (2.2) 25.1 (4.0) 24.3 (3.8)
						6 months	CHX 2% Control CHX 2% (CA) Control (CA)	31.3 (2.6) 24.2 (3.6) 23.2 (5.2) 14.3 (5.8)
Li et al/2018 48	BAI GD	120	Caries-free	Adper Single Bond 2 (etch and rinse)	Artificial saliva	24 hours	GD 5% BAI 2.5 μg/mL Control	58.86 (4.29) 58.32 (3.95) 41.89 (5.18)
						3 months	GD 5% BAI 2.5 μg/mL Control	56.10 (5.89) 52.43 (5.43) 34.46 (6.22)
						6 months	GD 5% BAI 2.5 μg/mL Control	51.86 (6.42) 52.43 (5.43) 26.82 (5.30)
Loguercio et al/2016 49	CHX	60	Caries-free	Primer & Bond NT (etch-and-rinse; PB) Adper Single Bond 2 (etch and rinse)	Water	24 hours	CHX 2% (PB) Control CHX 2% (ASB) Control	33.1 (2.8) 35.1 (3.1) 43.5 (3.5) 40.2 (3.3)
						5 years	CHX 2% (PB) Control CHX 2% (ASB) Control	22.1 (2.2) 11.0 (2.7) 31.3 (2.7) 16.1 (2.1)
Loguercio et al/2009 50	CHX	15/60	Caries-free	Primer & Bond (etch and rinse) Adper Single Bond (SB; etch and rinse)	Water	24 hours	CHX 2% (PB) 15 s CHX 0.002% (PB) 15 s Control 15 s CHX 2% (SB) 15 s CHX 0.002% (SB) 15 s Control 15 s CHX 2% (PB) 60 s CHX 0.002% (PB) 60 s Control 60 s CHX 2% (SB) 60 s CHX 0.002% (SB) 60 s Control 60 s	33.1 (6.5) 25.7 (2.4) 28.3 (4.3) 43.5 (4.1) 41.4 (4.8) 39.2 (5.4) 31.3 (5.1) 29.2 (3.4) 32.4 (5.4) 41.2 (4.2) 43.2 (6.1) 41.5 (6.4)
						6 months	CHX 2% (PB) 15 s CHX 0.002% (PB) 15 s Control 15 s CHX 2% (SB) 15 s CHX 0.002% (SB) 15 s Control 15 s CHX 2% (PB) 60 s CHX 0.002% (PB) 60 s Control 60 s CHX 2% (SB) 60 s CHX 0.002% (SB) 60 s Control 60 s	27.3 (4.2) 23.2 (4.1) 20.1 (4.2) 40.1 (5.7) 37.2 (6.1) 27.9 (6.2) 28.1 (4.4) 27.0 (3.6) 21.2 (3.8) 37.6 (3.3) 40.1 (3.7) 25.4 (4.1)
Maravic et al/2018 51	ACR	60	Caries-free	Adper Scotchbond 1XT (etch and rinse)	Artificial saliva	24 hours	ACR 0.01% Control	46.6 (3.1) 46.0 (4.9)
						12 months	ACR 0.01% Control	39.9 (3.3) 24.8 (2.4)
Mazzoni et al/2013 52	EDC	60	Caries-free	Optibond (OB) FL (etch and rinse) Scotchbond (SB) 1XT (etch and rinse)	Artificial saliva	24 hours	EDC 0.3M + OB Control EDC 0.3M + SB Control	44.5 (9.8) 43.3 (9.4) 38.8 (9.8) 40.5 (10.3)
						12 months	EDC 0.3M + OB Control EDC 0.3M + SB Control	41.2 (10.1) 33.1 (7.9) 32.5 (9.6) 24.8 (8.8)
Mazzoni et al/2018 53	EDC	60	Caries-free	Clearfil SE primer (self-etch) XP Bond (etch and rinse)	Artificial saliva	24 hours	EDC 0.3M + (Clearfil) Control EDC 0.3M + (XP bond) Control	30.1 (6.3) 32.8 (4.4) 36.5 (7.1) 37.6 (5.9)
						12 months	EDC 0.3M + (Clearfil) Control EDC 0.3M + (XP bond) Control	26 (8.0) 21.4 (5.7) 28.6 (6.4) 18.1 (4.9)
Mohamed et al/2020 54	CS	60	Caries-free	Universal Single Bond adhesive (self-etch)	Water	24 hours	CS 0.2% CS 2.5% Control	39.16 (38.62) 15.63 (14.64) 20.82 (21.43)
						3 months	CS 0.2% CS 2.5% Control	23.95 (25.08) 16.89 (17.79) 21.1 (21.03)
						6 months	CS 0.2% CS 2.5% Control	25.1 (25.73) 21.36 (20.94) 28.76 (28.15)
Mosallam et al/2018 55	GT MA MN	60	Caries-free	Tetric N-Bond Universal (etch and rinse)	Water	24 hours	GT 20 mg/mL (WE) GT 5 mg/mL (AE) MA 20 mg/mL (WE) MA 5 mg/mL (AE) MN 20 mg/mL (WE) MN 5 mg/mL (AE) Control	29.22 (6.29) 16.70 (5.30) 4.01 (1.92) 26.68 (5.81) 24.90 (6.74) 26.68 (5.81) 28.38 (6.68)
						1,000 thermocycles	GT 20 mg/mL (WE) GT 5 mg/mL (AE) MA 20 mg/mL (WE) MA 5 mg/mL (AE) MN 20 mg/mL (WE) MN 5 mg/mL (AE) Control	18.97 (6.66) 12.73 (6.63) 2.64 (2.27) 17.93 (4.82) 17.83 (6.57) 17.93 (4.82) 17.39 (1.71)
Mosallam et al/2019 56	MA MN	60	Caries-free	Scotch Bond Universal (etch and rinse)	Water	24 hours	MA 20 mg/mL (WE) MA 5 mg/mL (AE) MN 20 mg/mL (WE) MN 5 mg/mL (AE) Control	29.30 (7.31) 17.39 (1.63) 35.03 (5.24) 19.72 (8.82) 28.38 (6.68)
						1,000 thermocycles	MA 20 mg/mL (WE) MA 5 mg/mL (AE) MN 20 mg/mL (WE) MN 5 mg/mL (AE) Control	20.55 (8.85) 10.26 (8.28) 20.60 (5.97) 18.05 (7.84) 17.39 (1.71)
Ou et al/2018 57	CHX MMP8-I inhibitor	30	Caries-free	Adper Single Bond 2 (etch and rinse)	Water	24 hours	CHX 2% MMP8-I Control	42.14 (8.83) 55.29 (9.71) 47.18 (11.69)
						6 months	CHX 2% MMP8-I Control	41.83 (15.52) 54.70 (13.66) 39.06 (9.88)
						12 months	CHX 2% MMP8-I Control	39.92 (16.08) 54.29 (15.26) 35.82 (19.14)
Paulose and Fawzy/2018 58	EDC	60	Caries-free	Adper Scotchbond multipurpose (etch-and-rinse: SBM) Single bond Universal adhesive (etch and rinse)	Water	24 hours	EDC 0.3M + SBM Control EDC 0.3M -dry + SBU Control EDC 0.3M -wet + SBU Control	40.7 (9.3) 43.2 (8.1) 39.7 (5.3) 36.9 (8.7) 30.9 (5.7) 33.6 (6.1)
						12 months	EDC 0.3M + SBM SMP Control EDC 0.3 -dry + SBU Control EDC 0.3M -wet + SBU Control	30.8 (7.4) 22.3 (7.3) 26.7 (4.9) 18.8 (5.9) 11.2 (4.6) 13.7 (4.6)
Pedrosa et al/2018 59	CA	60	Caries-free	Adper Scotchbond multipurpose (etch-and-rinse) Clearfil SE bond (self-etch)	Water	24 hours	CA 0.05% (ASB) CA 0.1% (ASB) Control CA 0.05% (CSE) CA 0.1% (CSE) Control	34.40 (7.75) 36.58 (6.16) 40.67 (8.90) 23.47 (6.91) 25.73 (5.55) 31.74 (8.05)
						12 months	CA 0.05% (ASB) CA 0.1% (ASB) Control CA 0.05% (CSE) CA 0.1% (CSE) Control	26.97 (9.88) 22.88 (4.44) 25.24 (9.72) 24.20 (7.78) 26.21 (7.33) 25.99 (6.79)
Perote et al/2015 60	CHX EPE APE	60	Caries-free	Adper Single Bond 2 (etch and rinse)	Artificial saliva	24 hours	CHX 0.2% EPE 10% APE 10% Control	31.6 (7.0) 29.1 (6.9) 33.0 (6.7) 28.6 (5.3)
						6 months	CHX 0.2% EPE 10% APE 10% Control	26.5 (4.4) 23.1 (3.9) 25.1 (4.8) 24.0 (3.9)
Porto et al/2018 61	CHX QUE Res	60	Caries-free	Single Bond Universal (etch and rinse)	Water	24 hours	CHX 2% Que (μg mL ^1^ ) 100 250 500 1,000 Res (μg mL ^1^ ) 100 250 500 1,000 Que + Res (μg mL ^1^ ) 3:1 100 250 500 1,000 Que + Res 1:1 100 250 500 1,000 Que + Res 1:3 100 250 500 1,000 Control	27.78 (6.88) 32.06 (8.90) 27.51 (8.70) 31.21 (9.93) 31.30 (10.33) 18.81 (6.07) 23.90 (7.46) 23.74 (5.98) 20.11 (5.31) 27.40 (7.19) 19.33 (6.02) 28.44 (7.07) 31.38 (8.45) 18.78 (3.63) 23.93 (7.20) 23.29 (5.23) 19.10 (5.49) 22.73 (6.37) 20.83 (6.61) 25.99 (7.89) 23.76 (5.76) 23.62 (6.71)
						3 months	CHX 2% Que (μg mL ^1^ ) 100 250 500 1,000 Res (μg mL ^1^ ) 100 250 500 1,000 Que + Res (μg mL ^1^ ) 3:1 100 250 500 1,000 Que + Res 1:1 100 250 500 1,000 Que + Res 1:3 100 250 500 1,000 Control	30.68 (8.71) 25.29 (8.01) 34.68 (16.17) 42.37 (13.59) 37.40 (11.37) 31.03 (11.25) 37.90 (10.11) 29.77 (7.34) 26.18 (7.77) 30.48 (10.16) 35.38 (13.54) 31.14 (10.31) 32.32 (8.39) 37.13 (12.29) 32.80 (14.05) 32.36 (11.43) 28.13 (8.54) 28.56 (11.45) 30.82 (8.77) 26.55 (7.93) 31.66 (10.92) 26.47 (8.26)
Prasansuttiporn et al/2020 62	RA	5	Caries-affected	Clearfil SE Bond (self-etch)	Artificial saliva	24 hours	RA 100 μM Control	35.4 (5.5) 35.1 (5.3)
						12 months	RA 100 μM Control	34.2 (4.3) 30.3 (4.2)
Prasansuttiporn et al/2017 63	RA	5	Caries-free	Clearfil SE Bond (self-etch)	Artificial saliva	24 hours	RA 100 μM Control	54.8 (3.9) 55.2 (4.1)
						12 months	RA 100 μM Control	52.6 (4.7) 45.8 (4.0)
Ruksaphon and Pisol/2017 64	CHX RA	60	Caries-free	OptiBond FL (etch and rinse) OptiBond Solo (solo) (etch and rinse)	Artificial saliva	24 hours	CHX 2% + (solo) CHX 2% + (FL) RA 100 μM + (solo) RA 100 μM + (FL) Control (solo) Control (FL)	38.42 (8.04) 38.46 (7.82) 36.00 (8.04) 41.27 (6.76) 39.60 (7.50) 37.27 (8.45)
						3 months	CHX 2% + (solo) CHX 2% + (FL) RA 100 μM + (solo) RA 100 μM + (FL) Control (solo) Control (FL)	40.75 (7.12) 41.26 (5.51) 39.43 (10.12) 41.27 (6.76) 32.13 (7.32) 29.45 (8.12)
						6 months	CHX 2% + (solo) CHX 2% + (FL) RA 100 μM + (solo) RA 100 μM + (FL) Control (solo) Control (FL)	32.83 (6.82) 29.33 (6.66) 31.37 (10.24) 32.79 (7.37) 30.54 (8.05) 26.46 (6.39)
						12 months	CHX 2% + (solo) CHX 2% + (FL) RA 100 μM + (solo) RA 100 μM + (FL) Control (solo)) Control (FL)	22.85 (11.72) 27.82 (11.54) 28.98 (7.68) 28.04 (9.09) 3.10 (8.22) 3.91 (9.20)
Sacramento et al/2012 65	CHX	60	Caries-affected	Clearfil protect Bond (self-etch) Clearfil SE Bond (self-etch)	Water	24 hours	CHX 2% (SE) CHX 2% (PB) Control (SE) Control (PB)	12.39 (2.37) 14.60 (3.65) 12.28 (2.91) 16.24 (2.71)
						6 months	CHX 2% (SE) CHX 2% (PB) Control (SE) Control (PB)	2.88 (1.30) 3.09 (0.92) 2.95 (0.77) 2.32 (0.60)
						12 months	CHX 2% (SE) CHX 2% (PB) Control (SE) Control (PB)	1.76 (0.35) 2.34 (0.76) 1.36 (0.22) 1.11 (0.59)
Sadeghi et al/2017 66	CHX	60	Caries-free	Optibond Solo Plus (etch and rinse) Single Bond Universal (SBU; etch and rinse)	Water	1 week	CHX 0.2% + OSP Control CHX 0.2% +SBU Control	29.84 (5.43) 34.57 (8.22) 35.75 (8.58) 58.17 (10.25)
						6 months	CHX 0.2% + OSP Control CHX 0.2% +SBU Control	20.59 (5.52) 22.51 (3.55) 23.28 (3.90) 33.42 (7.04)
Santiago et al/2013 67	CHX EGCG	60	Caries-free	Adper Single Bond 2 (etch and rinse)	Water	24 hours	EGCG 0.02% EGCG 0.1% EGCG 0.5% CHX 2% Control	31.39 (7.82) 34.74 (9.14) 27.11 (7.78) 34.68 (7.30) 34.17 (7.75)
						6 months	EGCG 0.02% EGCG 0.1% EGCG 0.5% CHX 2% Control	31.75 (10.58) 35.99 (10.91) 31.18 (9.29) 31.62 (5.78) 27.67 (6.98)
Shen et al/2020 68	CHX	60	Caries-free	Single Bond 2 (etch and rinse)	Water	24 hours	CHX 2% Control	37.43 (5.29) 33.00 (3.95)
						6 months	CHX 2% Control	33.31 (3.28) 28.36 (4.01)
Venigalla et al/2016 69	RIBO EDC PAC	120	Caries-free	Adper Single Bond water wet bonding (etch and rinse) Ethanol wet bonding (etch and rinse)	Artificial saliva	24 hours	RIBO 0.1% + WWB EDC 1M +WWB PAC 6.5% +WWB Control RIBO 0.1% + EWB EDC 1M + EWB PAC 6.5% + EWB Control	46.94 (2.17) 45.14 (1.76) 41.71 (1.63) 31.76 (1.51) 52.12 (0.46) 47.50 (0.78) 44.38 (0.69) 41.61 (1.13)
						6 months	RIBO 0.1% + WWB EDC 1M +WWB PAC 6.5% +WWB Control RIBO 0.1% + EWB EDC 1M + EWB PAC 6.5% + EWB Control	45.14 (1.50) 42.58 (1.24) 34.30 (1.21) 23.96 (1.43) 51.80 (0.32) 45.27 (0.50) 41.90 (0.79) 37.37 (0.58)
Xu et al/2020 70	BAC PVPA PAC	30	Caries-free	Clearfil SE bond (self-etch)	Water	24 hours	MDP 5% + BAC 1% MDP 5% + PVPA 1,000 μm/mL MDP 5% + PAC 15% Control MDP 15% + BAC 1% MDP 15% + PVPA 1,000 μm/mL MDP 15% + PAC 15% Control	29.2 (6.6) 27.9 (4.1) 26.5 (6.9) 26.9 (5.8) 31.7 (4.0) 30.4 (6.7) 30.3 (3.5) 29.3 (3.8)
						12 months	MDP 5% + BAC 1% MDP 5% + PVPA 1,000 μm/mL MDP 5% + PAC 15% Control MDP 15% + BAC 1% MDP 15% + PVPA 1,000 μm/mL MDP 15% + PAC 15% Control	25.9 (5.2) 26.8 (6.3) 25.6 (4.7) 26.3 (6.2) 35.2 (6.1) 31.8 (5.3) 29.7 (3.6) 31.5 (6.4)
Kazemi-Yazdi et al/2020 71	CHX	60	Caries-free	Clearfil SE Bond (self-etch)	Water	24 hours	CHX 2% Control	14.58 (5.04) 18.00 (5.54)
						3,000 thermocycles	CHX 2% Control	14.36 (7.44) 16.71 (8.00)
Da Silva et al/2015 72	CHX	60	Caries-free	Single Bond 2 (etch and rinse) Ambar (etch and rinse)	Water	24 hours	CHX 2% (SB) Control CHX 2% (Ambar) Control	21.7 (6.7) 11.4 (3.6) 11.2 (5.9) 12.5 (7.6)
						15 days	CHX 2% (SB) Control CHX 2% (Ambar) Control	11.1 (3.6) 6.3 (2.5) 6.8 (4.2) 7.7 (3.6)
Zheng et al/2015 73	CHX GT FeSO _4_ Galardin	60	Caries-free	Optibond FL (etch and rinse) Clearfil SE Bond (self-etch)	Artificial saliva	9 months	CHX 2% (FL) GT 0.05% (FL) FeSO _4_ 1 mM (FL) Galardin 0.2 mM (FL) Control CHX 2% (SE) GT 0.05% (SE) FeSO _4_ 1 mM (SE) Galardin 0.2 mM (SE) Control	32.9 (11.3) 33.2 (14.0) 25.3 (10.5) 33.6 (10.5) 25.3 (11.8) 32.9 (11.3) 26.1 (14.2) 25.3 (10.5) 33.6 (14.1) 20.3 (13.6)
Sadek et al/2010 74	CHX	60	Not mentioned	Scotchbond multipurpose (self-etch) Single Bond 2 (self-etch) Experimental ethanol wet-bonding adhesive (self-etch)	Artificial saliva	24 hours	CHX 2% + EWB Control CHX 2% + MP Control CHX 2% + SB Control	46.8 (5.1) 45.8 (7.2) 41.3 (8.1) 44.2 (3.5) 42.6 (5.2) 42.3 (7.4)
						9 months	CHX 2% + EWB Control CHX 2% + MP Control CHX 2% + SB Control	44.6 (5.6) 44.4 (6.9) 37.4 (5.6) 37.4 (3.5) 38.2 (4.7) 44.4 (4.9)
						18 months	CHX 2% + EWB Control CHX 2% + MP Control CHX 2% + SB Control	43.6 (5.5) 44.2 (7.8) 30.5 (8.0) 32.6 (7.1) 28.8 (8.3) 31.5 (4.3)
Breschi et al/2010 22	Galardin	30	Caries-free	Adper Scotchbond 1XT (etch and rinse)	Artificial saliva	24 hours	Galardin 0.2 mM Control	44.1 (7.3) 41.4 (5.9)
						12 months	Galardin 0.2 mM Control	32.4 (6.6) 22.6 (5.4)
Stanislawczuk et al/2009 75	CHX	60	Caries-free	Prime & Bond NT (etch and rinse) Single Bond (SB) 2 (etch and rinse)	Water	24 hours	CHX 2% + Prime & Bond Control CHX 2% + (SB) Control	21.9 (4.7) 22.0 (9.7) 23.4 (2.1) 14.6 (3.1)
						6 months	CHX 2% + Prime & Bond Control CHX 2% + (SB) Control	31.1 (3.1) 27.2 (6.1) 31.1 (2.6) 20.4 (2.1)
Firouzmandi et al/2020 76	SDF	180	Caries-free and Caries-affected (CA)	Adper single Bond 2 (etch and rinse)	Water	24 hours	SDF 30% Control SDF 30% (CA) Control	17.08 (4.88) 18.37 (4.71) 17.63 (4.19) 12.20 (2.34)
						6 months	SDF 30% Control SDF 30% (CA) Control	15.72 (2.34) 14.72 (3.51) 10.30 (3.78) 11.53 (2.66)
Giacomini et al/2017 77	CHX E-64	60	Caries-free Eroded (ERO) and Caries-affected (CA)	Adper Single Bond Universal (etch and rinse)	Artificial saliva	24 hours	CHX 2% CHX 2% (ERO) CHX 2% (CA) E-64 5 μM E-64 5 μM (ERO) E-64 5 μM (CA) Control Control (ERO/water) Control (CA/water)	28.36 (5.88) 22.53 (4.76) 18.31 (3.50) 28.33 (5.42) 30.23 (6.51) 24.51 (4.41) 35.32 (5.30) 29.85 (4.77) 23.42 (4.95)
						6 months	CHX 2% CHX 2% (ERO) CHX 2% (CA) E-64 5 μM E-64 5 μM (ERO) E-64 5 μM (CA) Control Control (ERO/water) Control (CA/water)	16.50 (3.89) 20.13 (4.62) 16.50 (3.90) 20.80 (3.71) 27.70 (5.32) 20.80 (3.71) 27.45 (5.33) 26.07 (4.96) 20.28 (3.55)
Sabatini et al/2014 78	CHX BAC	60	Caries-free	Adper Single Bond Plus (etch and rinse)	Artificial saliva	24 hours	CHX 2% BAC 0.5% BAC 1.0% Control	38.3 (10.3) 36.4 (8.4) 51.4 (7.9) 34.3 (7.8)
						6 months	CHX 2% BAC 0.5% BAC 1.0% Control	34.3 (5.2) 36.6 (6.2) 53.9 (6.9) 27.4 (6.2)
Carvalho et al/2016 79	CHX EGCG	60	Caries-affected	Adper Single Bond 2 (etch-and-rinse)	Water	24 hours	EGCG 2% CHX 2% Control	23.0 (6.3) 23.3 (6.0) 24.3 (8.6)
						6 months	EGCG 2% CHX 2% Control	35.7 (8.4) 23.0 (7.2) 21.6 (6.4)
Loguercio et al/2016 80	CHX	15	Caries-free	Prime & Bond NT (etch and rinse) Adper Single Bond 2 (etch and rinse)	Water	24 hours	CHX 2% (PB) Control CHX 2% (SB) Control	44.2 (4.3) 42.3 (3.4) 50.3 (5.6) 46.2 (4.7)
						2 years	CHX 2% (PB) Control CHX 2% (SB) Control	36.3 (5.1) 23.6 (5.3) 43.3 (3.5) 32.3 (4.5)
Cova et al/2011 99	RIBO	60	Caries-free	XP Bond adhesive (etch and rinse)	Artificial saliva	24 hours	RIBO 0.1% Control	44.4 (10.4) 37.3 (10.3)
						6 months	RIBO 0.1% Control	35.6 (11.2) 22.0 (7.0)
						12 months	RIBO 0.1% Control	30.9 (12.2) 17.7 (9)
Mobarak/2011 81	CHX	60	Caries-free and Caries-affected (CA)	Self-etch primer adhesive (Clearfil SE Bond; self-etch)	Artificial saliva	24 hours	CHX 2% CHX 5% Control CHX 2% (CA) CHX 5% (CA) Control	23.79 (5.9) 25.94 (6.4) 24.33 (5.1) 20.84 (6.2) 20.59 (5.1) 21.73 (6.0)
						2 years	CHX 2% CHX 5% Control CHX 2% (CA) CHX 5% (CA) Control	8.74 (3.2) 10.98 (3.3) 9.46 (3.4) 9.99 (3.4) 14.67 (4.5) 9.97 (3.5)
Manso et al/2014 82	CHX	30	Caries-free	All Bond 3 (Bisco) (etch and rinse) Excite (Ivoclar Vivadent) (etch and rinse)	Water	24 hours	CHX 2%/water (Bisco) Control CHX 2%/ethanol (Bisco) Control CHX 2%/water (Excite) Control CHX 2%/ethanol (Excite) Control	46.96 (3.6) 51.07 (3.6) 54.67 (3.6) 59.41 (3.6) 40.05 (5.4) 49.51 (5.4) 53.37 (5.4) 49.67 (5.4)
						6 months	CHX 2%/water (Bisco) Control CHX 2%/ethanol (Bisco) Control CHX 2%/water (Excite) Control CHX 2%/ethanol (Excite) Control	50.69 (3.6) 57.13 (3.6) 52.17 (3.6) 56.41 (3.6) 36.78 (5.4) 42.10 (5.4) 57.47 (5.4) 44.56 (5.4)
						15 months	CHX 2%/water (Bisco) Control CHX 2%/ethanol (Bisco) Control CHX 2%/water (Excite) Control CHX 2%/ethanol (Excite) Control	46.07 (4.4) 47.29 (4.4) 39.58 (4.4) 44.41 (4.4) 40.87 (6.6) 45.51 (6.6) 49.55 (6.6) 42.48 (5.4)
Breschi et al/2010 19	CHX	30	Caries-free	Adper Scotchbond 1XT (etch and rinse)	Artificial saliva	24 hours	CHX 2% CHX 0.2% Control	41.2 (9.6) 39.2 (9.3) 40.8 (8.7)
						2 years	CHX 2% CHX 0.2% Control	28.5 (7.2) 32.6 (8.3) 13.4 (4.9)
Montagner et al/2015 83	CHX	60	Caries-free	Adper Single Bond 2 (etch and rinse)	Water	24 hours	CHX 2% Control	25.3 (6.2) 26.7 (10.0)
						18 months	CHX 2% Control	20.1 (10.3) 14.8 (9.4)
Li et al/2020 84	DMA	60	Caries-free	Adper Single Bond 2 (etch and rinse)	Water	24 hours	DMA 0.1 mM DMA 1.0 mM DMA 10 mM Control	28.73 (5.19) 30.76 (7.57) 27.06 (7.53) 29.96 (6.43)
						1,000 thermocycles	DMA 0.1 mM DMA 1.0 mM DMA 10 mM Control	23.84 (7.06) 29.19 (6.58) 23.34 (7.36) 16.24 (6.90)
Hass et al/2016 98	PAC RIBO GD	60	Caries-free	Adper Single Bond 2 (etch and rinse) Tetric N-Bond (etch and rinse)	Water	24 hours	PAC 6.5% (SB) RIBO 0.1% (SB) GD 5% (SB) Control PAC 6.5% (TN) RIBO 0.1% (TN) GD 5% (TN) Control	36.2 (5.5) 37.1 (9.7) 38.5 (2.4) 39.5 (7.9) 29.2 (1.2) 31.5 (6.9) 35.7 (1.9) 36.8 (4.7)
						18 months	PAC 6.5% (SB) RIBO 0.1% (SB) GD 5% (SB) Control PAC 6.5% (TN) RIBO 0.1% (TN) GD 5% (TN) Control	31.9 (4.3) 31.6 (3.5) 29.7 (2.6) 13.9 (1.8) 27.6 (6.3) 25.1 (1.3) 24.2 (1.4) 13.9 (1.8)
Kalagi et al/2020 85	CHX	5	Caries-free	Adper Scotchbond multipurpose (etch and rinse)	Water	24 hours	CHX 2% Control	66.4 (8.8) 49.1 (12.6)
						6 months	CHX 2% Control	71.9 (14.7) 41.6 (10.6)
Tekçe et al/2016 86	CHX	60	Caries-free	Single Bond Universal (self-etch) All Bond Universal (self-etch)	Water	24 hours	CHX 2% (SBU) Control CHX 2% (ABU) Control	45.22 (6.32) 43.33 (3.41) 38.92 (4.01) 43.81 (3.61)
						12 months	CHX 2% (SBU) Control CHX 2% (ABU) Control	41.19 (3.98) 37.67 (3.40) 31.37 (5.97) 38.54 (6.19)
de Moura et al/2021 87	GT	60	Caries-affected	Adper Single Bond 2 (etch-and-rinse)	Water	24 hours	GT 0.05% GT 0.2% GT 2% Control	14.42 (6.20) 17.80 (6.49) 11.04 (2.94) 11.29 (4.78)
						6 months	GT 0.05% GT 0.2% GT 2% Control	9.53 (4.83) 13.25 (5.82) 7.09 (4.14) 8.82 (6.23)
Li et al/2021 88	DMA	60	Caries-free	Adper Single Bond 2 (etch-and-rinse)	Water	24 hours	DMA 1 mM DMA 5 mM DMA 10 mM Control	33.16 (8.41) 32.59 (8.70) 32.73 (7.39) 30.08 (7.55)
						10,000 thermocycles	DMA 1 mM DMA 5 mM DMA 10 mM Control	30.40 (8.10) 31.46 (7.31) 31.85 (8.10) 22.63 (6.40)

Abbreviations: ACR, acrolein; AE, alcohol extract; APE, aqueous propolis extract; BAI, baicalein; BAC, benzalkonium chloride; CA, caffeic acid; CS, chitosan; CHX, chlorhexidine; DCC, N,N'-dicyclohexylcarbodiimide; DMA, dopamine methacrylamide; EDC, carbodiimide; EGCG, epigallocatechin gallate; EPE, ethanolic propolis extract; FeSO _4_ , ferrous sulfate; GD, 5% glutaraldehyde; GT, green tea; HES, hesperidin; MA, *Morus alba* leaves; MAP, mussel adhesive protein; MN, *Morus nigra* leaves; NAR, naringin; PAC, proanthocyanidin; PVPA, polyvinylphosphonic acid; QUE, guercetin; RA, rosmarinic acid; Res, resveratrol; RIBO, riboflavin; RUT, rutin; SDF, silver diamine fluoride; WE, water extract.

Thirty-one different types of MMP inhibitors were used, 14 synthetically derived and 17 naturally derived. The microtensile bond strength test was used in all included studies except for five studies that used microshear bond strength testing. Most studies ( *n* = 53) used caries-free dentine substrate, 13 used caries-affected dentine, two studies used eroded dentine, and one study used dentine without mentioning its condition. All studies used permanent teeth except for one study that used primary teeth.

With respect to storage medium, the majority of studies used distilled water (40 studies) and 22 used artificial saliva. Two studies used both distilled and deionized water. The majority of the studies applied MMP inhibitor for 60 s ( *n* = 47), six studies applied it for 30 seconds, four for 120 seconds, three for 5 seconds, two for 15 seconds, and one each for 20 and 180 seconds. One study did not report the application duration. Only MMP inhibitors applied for 30 and 60 seconds were included in the meta-analysis, as they contained enough data.

Ageing periods ranged from 24 hours to 5 years, and various thermocycling ageing protocols were also used. The majority of studies ( *n* = 62) aged samples for 24 hours as an immediate ageing period. With respect to long-term ageing, 31 studies aged the samples for 6 months, 19 aged them for 12 months, five aged them for 3 months, three for 2 years, three for 18 months, two for 9 months, and one study each for 3 days, 1 week, 15 days, 15 months, and 5 years. Eleven studies used thermocycling for ageing: four used 1,000 cycles, two used 5,000 cycles, and one study each used 2,500, 3,000, 10,000, and 25,000 cycles.

### Risk of Bias Evaluation

Table 3 shows the evaluated risk of bias of the included studies. Overall, almost half of included studies showed a medium risk of bias (33 of 64), 17 of 64 studies showed a high risk of bias, and 14 studies were classified as a low risk of bias.

**Table 3:** Quality assessment and risk of bias

Study/year	Randomization	Substrate condition	Dentine pretreatment duration	Manufacturer instruction	Storage medium	Interface surface area	Single operator	Sample size calculation	Blinding of operator	Risk of bias
**Baena et al** / **2020** 30	N	Y	Y	Y	Y	Y	N	N	N	High
**Balloni et al** / **201** 7 31	Y	Y	Y	Y	Y	Y	N	N	Y	Medium
**Bravo et al** / **2017** 32	Y	Y	Y	Y	Y	Y	N	N	N	Medium
**de Faria Teixeira et al** / **2015** 33	Y	N	Y	Y	Y	Y	N	N	N	High
**Comba et al** / **2020** 34	Y	Y	Y	Y	Y	Y	N	N	N	Medium
**Czech et al** / **2019** 24	Y	Y	Y	Y	Y	Y	Y	N	N	Medium
**Dávila-Sánchez et al** / **2020** 35	Y	Y	Y	Y	Y	Y	Y	Y	N	Low
**Costa et al** / **2019** 36	Y	Y	Y	Y	Y	Y	N	N	N	Medium
El Baz, and Aboulenien/2018 37	N	Y	Y	Y	Y	Y	N	Y	N	Medium
**Fang et al** / **2017** 38	N	Y	Y	Y	Y	Y	N	N	N	High
**Fernandes et al** / **202** 1 39	Y	Y	Y	Y	Y	Y	N	Y	N	Medium
**Fialho et al** / **2019** 40	Y	Y	Y	Y	Y	Y	Y	Y	N	Low
**Gerhardt et al** / **2016** 41	Y	Y	Y	Y	Y	N	N	N	N	High
**Campos et al** / **2019** 42	Y	Y	N	Y	Y	Y	Y	N	N	Medium
**Giacomini et al** / **2020** 43	Y	Y	Y	Y	Y	Y	N	Y	N	Medium
Grandizoli and Pinheiro/2018 44	Y	Y	Y	Y	Y	Y	N	Y	N	Medium
**Karrabi** and Danesh Kazemi/ **2016** 45	Y	Y	Y	Y	Y	Y	N	N	N	Medium
**Kasraei** et al/ **2017** 46	Y	Y	Y	N	Y	Y	N	N	N	High
**Lenzi et al** / **2014** 47	Y	Y	Y	N	Y	Y	N	N	N	High
**Li et al** / **2018** 48	Y	Y	Y	N	Y	Y	N	N	N	High
**Loguercio et al** / **2016** 49	Y	Y	Y	N	Y	Y	N	N	N	High
**Loguercio et al** / **2009** 50	Y	Y	Y	Y	Y	Y	Y	N	N	Medium
**Maravic et al** / **2018** 51	Y	Y	Y	Y	Y	Y	N	N	N	Medium
**Mazzoni et al** / **2013** 52	Y	Y	Y	Y	Y	Y	N	N	N	Medium
**Mazzoni et al** / **2018** 53	Y	Y	Y	Y	Y	Y	N	N	N	Medium
**Mohamed et al** / **2020** 54	N	Y	Y	Y	Y	Y	N	N	N	High
**Mosallam et al** / **2018** 55	Y	Y	Y	Y	Y	Y	N	N	N	Medium
**Mosallam et al** / **2019** 56	Y	Y	Y	Y	Y	N	N	N	N	High
**Ou et al** / **2018** 57	Y	Y	Y	Y	Y	N	N	N	N	High
**Paulose and Fawzy** / **2018** 58	Y	Y	Y	Y	Y	Y	N	N	N	High
**Pedrosa et al** / **2018** 59	Y	Y	Y	Y	Y	Y	N	N	N	Medium
**Perote et al** / **2015** 60	Y	Y	Y	Y	Y	Y	N	N	N	Medium
**Porto** et al/ **2018** 61	Y	Y	Y	Y	Y	Y	N	N	N	Medium
**Prasansuttiporn et al** / **2020** 62	Y	Y	Y	Y	Y	Y	N	N	N	Medium
**Prasansuttiporn et al** / **2017** 63	Y	Y	Y	Y	Y	Y	N	N	N	Medium
**Ruksaphon** and Pisol/ **2017** 64	Y	Y	Y	Y	Y	Y	N	N	N	Medium
**Sacramento et al** / **2012** 65	Y	Y	Y	Y	Y	Y	N	N	N	Medium
**Sadeghi** et al/ **2017** 66	Y	Y	Y	Y	Y	Y	N	N	N	Medium
**Santiago et al** / **2013** 67	Y	Y	Y	Y	Y	Y	N	N	N	Medium
**Shen et al** / **2020** 68	Y	Y	Y	N	Y	Y	N	N	N	High
**Venigalla et al** / **2016** 69	Y	Y	Y	Y	Y	N	N	N	N	High
**Xu et al** / **2020** 70	Y	Y	Y	N	Y	Y	N	N	N	High
**Kazemi-Yazdi et al** / **2020** 71	Y	Y	Y	Y	Y	Y	N	N	N	Medium
**Da Silva et al** / **2015** 72	Y	Y	Y	Y	Y	Y	N	N	N	Medium
**Zheng et al** / **2015** 73	Y	Y	Y	Y	Y	Y	N	N	N	Medium
**Sadek et al** / **2010** 74	Y	N	Y	Y	Y	Y	N	N	N	High
**Breschi et al** / **2010** 22	Y	Y	Y	Y	Y	Y	N	N	N	Medium
**Stanislawczuk et al** / **2009** 75	Y	Y	Y	N	Y	Y	Y	N	N	Medium
**Firouzmandi et al** / **2020** 76	N	Y	Y	Y	Y	N	N	N	N	High
**Giacomini et al** / **2017** 77	Y	Y	Y	Y	Y	Y	N	N	N	Medium
**Sabatini et al** / **2014** 78	Y	Y	Y	Y	Y	Y	N	N	N	Medium
**Carvalho et al** / **2016** 79	Y	Y	Y	Y	Y	Y	N	N	N	Medium
**Loguercio et al** / **2016** 80	Y	Y	Y	Y	Y	Y	Y	N	N	Medium
**Cova et al** / **2011** 99	Y	Y	Y	Y	Y	Y	N	N	N	Medium
**Mobarak** / **2011** 81	N	Y	Y	Y	Y	N	N	N	N	High
**Manso et al** / **2014** 82	Y	Y	Y	Y	Y	Y	N	N	N	Medium
**Breschi et al** / **2010** 19	Y	Y	Y	Y	Y	Y	N	N	N	Medium
**Montagner et al** / **2015** 83	Y	Y	Y	Y	Y	Y	Y	N	N	Medium
**Li et al** / **2020** 84	Y	Y	Y	Y	Y	Y	N	N	N	Medium
**Hass et al** / **2016** 98	Y	Y	Y	Y	Y	Y	N	N	N	Medium
**Kalagi et al** / **2020** 85	Y	Y	Y	Y	Y	Y	N	N	N	Medium
Tekçe **et al** / **2016** 86	Y	Y	Y	Y	Y	Y	Y	N	N	Medium
**de Moura et al** / **2021** 87	Y	Y	Y	Y	Y	Y	Y	Y	N	Low
**Li et al** / **2021** 88	Y	Y	Y	Y	Y	Y	N	N	N	Medium

Abbreviations: N, no; Y, yes.

Note: This table demonstrates the quality assessment and risk of bias as reported in the materials and methods section.

## Meta-Analysis

**Fig. 2:**
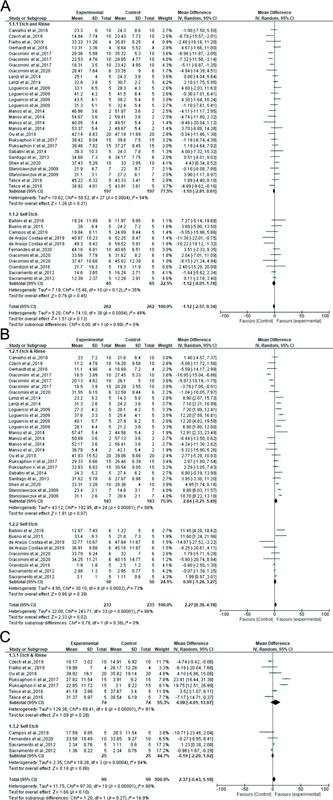
Forest plots according to MMP inhibitor type. 2% CHX vs. control at 24 hours ( **A** ), 6 months ( **B** ), and 12 months ( **C** ). CHX, chlorhexidine; CI, confidence interval; MMP, matrix metalloproteinase; SD, standard deviation.

Of the 64 studies, data from 42 studies were subjected to further evaluation in meta-analyses ( Figs. 2 3 4 5 6 ). In the first analysis (2% CHX vs. control in the baseline, immediate bond strength values), 16 etch-and-rinse studies were included, representing 28 datasets considered. There was no statistically significant difference between groups ( *Z* -test = 1.26, *p* = 0.21), and there was considerable heterogeneity ( *I ^2^* = 54%). Eight self-etching studies were included, with 11 datasets considered. There was no significant difference between groups ( *Z* -test = 0.76, *p* = 0.45), and there was moderate heterogeneity ( *I ^2^* = 35%). Overall (self-etching and etch-and-rinse), there was no statistically significant difference between groups ( *Z* -test = 1.51, *p* = 0.13), with moderate heterogeneity observed between subgroups ( *I ^2^* = 49%; Fig. 2A ).

**Fig. 3:**
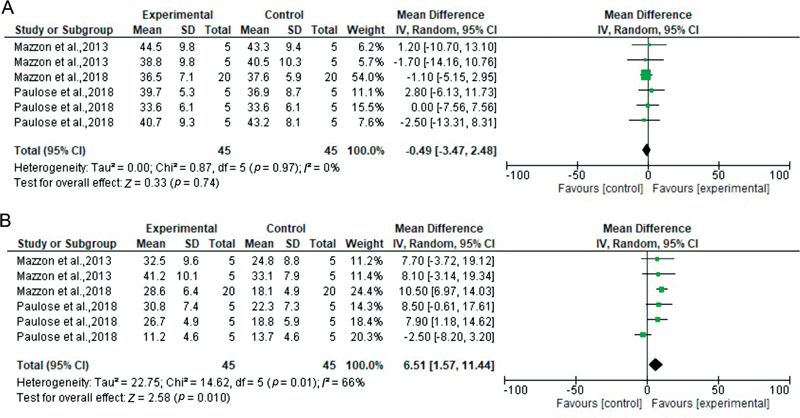
Forest plots according to MMP inhibitor type. 0.3 M EDC vs. control at 24 hours ( **A** ) and 12 months ( **B** ). CI, confidence interval; EDC, carbodiimide; MMP, matrix metalloproteinase; SD, standard deviation.

**Fig. 4:**
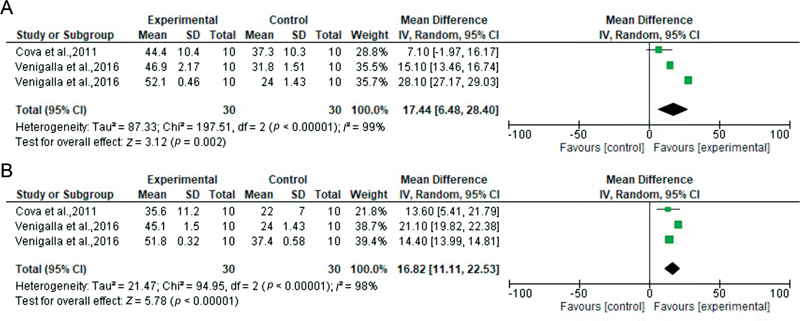
Forest plots according to MMP inhibitor type: 0.1% RIBO vs. control at 24 hours ( **A** ) and 6 months ( **B** ). CI, confidence interval; MMP, matrix metalloproteinase; RIBO, riboflavin; SD, standard deviation.

**Fig. 5:**
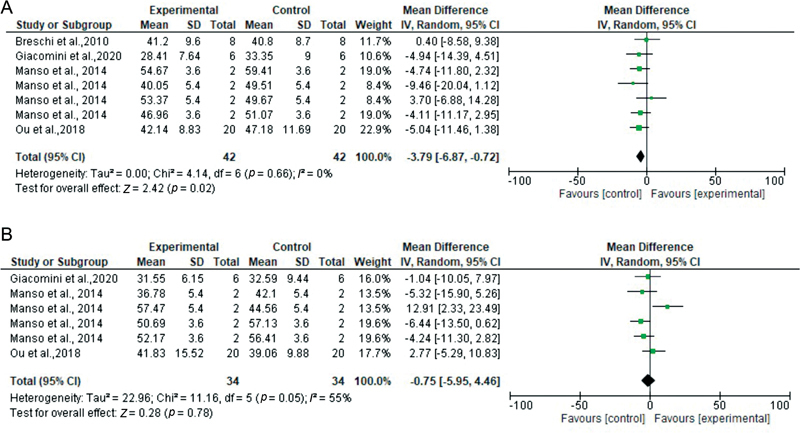
Forest plots according to pretreatment duration for 30 seconds: pretreatment with 2% CHX vs. control group at 24 hours ( **A** ) and 6 months ( **B** ). CHX, chlorhexidine; CI, confidence interval; SD, standard deviation.

**Fig. 6:**
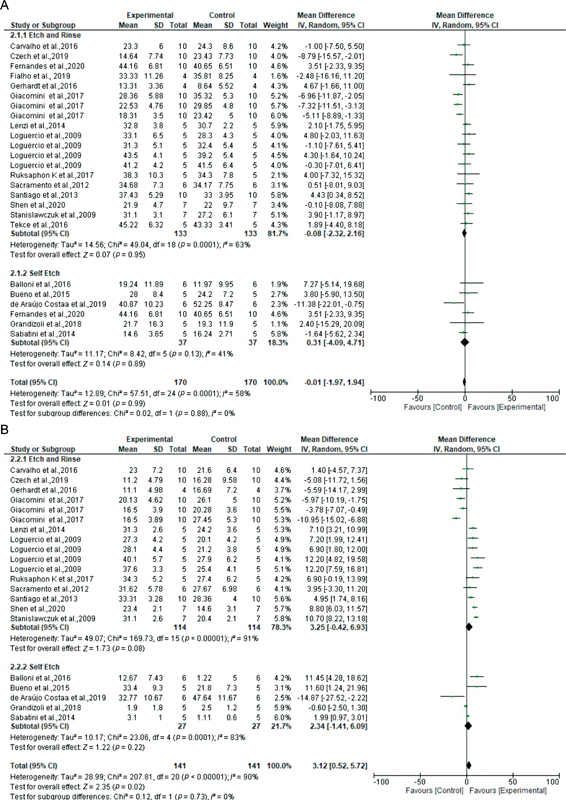
Forest plots according to pretreatment duration for 60 seconds: pretreatment with 2% CHX vs. control group at 24 hours ( **A** ) and 6 months ( **B** ). CHX, chlorhexidine; CI, confidence interval; SD, standard deviation..

The second analysis (2% CHX vs. control at 6 months of ageing) included 14 etch-and-rinse studies, representing 25 datasets. There was overall a higher bond strength for the experimental group compared with controls, but this was not statistically significant ( *Z* -test 1.81, *p* = 0.07) and heterogeneity was considerable ( *I ^2^* = 88%). Six self-etching studies were included, with nine datasets considered. There was no statistically significant difference between groups ( *Z* -test = 0.86, *p* = 0.39), and again there was considerable heterogeneity ( *I ^2^* = 73%). Tests for overall effect showed significantly higher bond strength in the experimental group compared with controls ( *Z* -test = 2.33, *p* = 0.02), with considerable heterogeneity between subgroups ( *I ^2^* = 86%; Fig. 2B ).

The third analysis (2% CHX vs. control at 12 months of ageing) included five etch-and-rinse studies with seven datasets. There were overall higher bond strength values in the experimental group compared with the control group. but this was not statistically significant ( *Z* -test = 1.09, *p* = 0.28) and heterogeneity was considerable ( *I ^2^* = 91%). For self-etching, three studies were included with four datasets considered, and there was no statistically significant difference between groups ( *Z* -test = 0.18, *p* = 0.86) but with considerable heterogeneity ( *I ^2^* = 84%). Tests for overall effect favored the experimental group over the control group but without statistical significance ( *Z* -test = 1.66, *p* = 0.10) and with considerable heterogeneity between subgroups ( *I ^2^* = 90%; Fig. 2C ).

For the fourth analysis (0.3 EDC vs. control at baseline), only etch-and-rinse studies met the inclusion criteria. Three studies were included, representing six datasets. Overall, the effect was not statistically significant ( *Z* -test = 0.33, *p* = 0.74). Heterogeneity between groups was low ( *I ^2^* = 0%; Fig. 3A ).

For the fifth analysis (0.3 EDC vs. control at 12 months), again, three etch-and-rinse studies representing six datasets were included. Overall, there were significantly higher bond strength values in the experimental group compared with the control group ( *Z* -test = 2.58, *p* = 0.01) but with considerable heterogenicity ( *I* ^2^ = 66%; Fig. 3B ).

For the sixth analysis (0.1% RIBO vs. control at baseline), only two etch-and-rinse studies met the criteria, representing three datasets. There was overall a significant difference favoring the experimental group over the control group ( *Z* -test = 3.12, *p* = 0.002), with considerable heterogeneity ( *I ^2^* = 99%; Fig. 4A ).

For the seventh analysis (0.1% RIBO vs. control at 6 months), two studies representing three datasets showed significantly higher bond strengths in the experimental group than the control group ( *Z* -test = 5.78, *p* < 0.00001) but with considerable heterogeneity *I ^2^* = 98% ( Fig. 4B ).

For the eighth analysis of pretreatment for 30 seconds (2% CHX vs. control at baseline), only four etch-and-rinse studies were included, representing seven datasets. There was overall a statistically significant difference favoring the control group over the experimental group ( *Z* = 2.42, *p* = 0.02), and heterogeneity was low ( *I ^2^* = 0%; Fig. 5A ).

For the ninth analysis of pretreatment for 30 seconds (2% CHX vs. control at 6 months), only three etch-and-rinse studies met the criteria, representing six datasets. There was overall no statistically significant difference between groups ( *Z* = 0.28, *p* = 0.78), and heterogeneity was considerable ( *I ^2^* = 55%; Fig. 5B ).

For the 10th analysis of pretreatment for 60 seconds (2% CHX vs. control at baseline), 14 etch-and-rinse studies were included, representing 19 datasets. There was overall no statistically significant difference between groups ( *Z* -test = 0.07, *p* = 0.95), but there was considerable heterogeneity between groups ( *I ^2^* = 63%). For self-etching, six studies were included with six datasets. Again, there was no statistically significant difference between groups ( *Z* -test = 0.01, *p* = 0.89) and moderate heterogeneity ( *I ^2^* = 41%). Tests for overall effect showed no statistically significant difference between groups ( *Z* -test = 0.01, *p* = 0.99) and considerable heterogeneity between subgroups ( *I ^2^* = 58%; Fig. 6A ).

For the 11th and final analysis of pretreatment for 60 seconds (2% CHX vs. control at 6 months), 11 etch-and-rinse studies were included, representing 16 datasets. Overall, the experimental group was slightly, but not significantly, favored over the control group ( *Z* -test = 1.73, *p* = 0.08), with considerable heterogeneity ( *I ^2^* = 91%). Five self-etching studies were included representing five datasets. Overall, the experimental group was slightly, but not significantly, favored over the control group ( *Z* -test = 1.22, *p* = 0.22), with considerable heterogeneity ( *I ^2^* = 83%). The tests for overall effect favored the experimental group but this was not statistically significant ( *Z* -test = 2.35, *p* = 0.73). Heterogeneity between subgroups was considerable ( *I ^2^* = 90%; Fig. 6B ).

## Discussion

This meta-analysis revealed that at least some MMP inhibitors significantly alter bond strength, both immediately and over the longer term. Accordingly, the null hypothesis was rejected.

Of all MMP inhibitors considered for meta-analysis, two MMP inhibitors improved bond strength: 0.3 M EDC and 0.1% RIBO. The 0.3 M EDC did not improve bond strength immediately (24 hours) but showed benefit after ageing for 12 months, while 0.1% RIBO showed statistically significant increases in bond strength both immediately (24 hours) and over the long term (6 months) compared with controls. Conversely, 2% CHX showed a slight but nonsignificant improvement in bond strength after 6 months of ageing but not immediately (24 hours) or after 12 months. The lack of immediate benefit with 2% CHX is consistent with two previous meta-analyses, 28 89 but the long-term results differ, possibly due to the different concentration of CHX used in previous studies. It has been suggested but not consistently proven that MMP inhibition by CHX is dose dependent. 90 91 It is worth noting that, of the few clinical trials evaluating pretreatment with CHX, no improvement in bond strength was observed over time. 92 93 94 95 96 97 With respect to adhesive systems, a previous systematic review 28 found that both types of adhesive system (self-etching and etch and rinse) benefited from 2% CHX *in vitro* . This, however, was also not consistent with the current meta-analysis results, since we found no significant difference according to the adhesive system used.

EDC and RIBO have a different mechanism of MMP inhibition to CHX through their cross-linking action. Generally, collagen cross-linkers protect collagen fibrils from further degradation by enhancing both the chemical and mechanical properties of collagen. 98 99 100 These additional functions could explain their superiority in maintaining adhesive interface integrity.

Pretreatments of 30 and 60 seconds with 2% CHX met the inclusion criteria for meta-analysis. Generally, neither pretreatment protocol significantly improved bond strength either immediately (24 hours) or over the long term (6 months). Indeed, when 2% CHX was applied for 30 seconds, there was a significant negative effect on bond strength over 24 hours. After 6 months of aging, there was a slight improvement in bond strength, still favoring the control group. With pretreatments of 60 seconds, 2% CHX showed no effect on bond strength and was similar to controls and, while slightly improved bond strength was observed with CHX after 6 months, it was nevertheless not statistically significant.

Our results show some inconsistencies with previous systematic reviews which might be due to differences in the inclusion criteria. For example, Montagner et al 28 and Kiuru et al 89 included different concentrations of CHX other than 2%, as well as various bond strength tests other than microtensile bond strength testing.

## Limitations

There are a few limitations to our study. This review only included *in vitro* studies since there have been very few *in vivo* studies or clinical trials in the literature. More *in vivo* studies will ultimately be crucial for providing high-quality evidence of the safety, toxicity, and efficacy of a given intervention in a complex model. Furthermore, although strict measures were taken during the search of the articles included for meta-analysis, several data demonstrated high heterogeneity. It is worth mentioning that most of the results with high heterogeneity were observed in the long-term ageing periods, unlike the immediate ageing periods which showed lower heterogeneity. Factors that could influence this may include the different brands of adhesive systems used and the ageing methods utilized. Similar findings were observed in the study by Montagner et al 28 which found that the aging methods were the greater influencing factor in the high heterogeneity. It is also worth noting that there are no standardized protocols for evaluating bond strength which previously shown will inevitably increase the heterogeneity of results 101 . To improve the reliability and quality of future bond strength testing studies, robust and strict guidelines for laboratory testing must be developed and implemented.

Many of the studies carried a risk of bias, and only one study mentioned blinding of the operator testing the bond strength; this parameter will be important to include in future studies to reduce the risk of bias. Moreover, only six studies calculated the sample size and reported a power analysis.

Nevertheless, these *in vitro* findings pave the way for rationale clinical trialing of dentine surface pretreatment with MMP inhibitors to improve clinical outcomes.

## Conclusion

The data suggest that using 2% CHX had no significant positive effect on bond strength either immediately or over the longer term. Pretreatments with 2% CHX for either 30 or 60 seconds do not improve the bond strength. Both 0.3 M EDC and 0.1% RIBO improve bond strength immediately and over time. There was considerable heterogeneity between the different adhesive systems used, limiting our meta-analysis. Given the limited clinical evidence available, more research is required to confirm the beneficial use of MMP inhibitors.
